# Measuring Peripheral Tissue DHA Turnover Using a Novel ^13^C Enrichment Technique

**DOI:** 10.1002/lipd.70011

**Published:** 2025-10-03

**Authors:** Brinley J. Klievik, Adam H. Metherel, Rodrigo Valenzuela, Richard P. Bazinet

**Affiliations:** ^1^ Department of Nutritional Sciences, Temerty Faculty of Medicine University of Toronto Toronto Ontario Canada; ^2^ Department of Nutrition, Faculty of Medicine University of Chile Santiago Chile

**Keywords:** diet, docosahexaenoic acid, fatty acid metabolism, heart, isotope ratio mass spectrometry, muscle, n‐3 polyunsaturated fatty acids, perirenal adipose, red blood cells, skin

## Abstract

Recently, through the use of compound‐specific isotope analysis (CSIA), our lab validated the utility of ^13^C enrichment (*δ* ^13^C) of docosahexaenoic acid (DHA) by using a very high *δ* ^13^C in a diet switch study by measuring brain, liver, and plasma DHA turnover and half‐lives via high‐precision gas chromatography combustion isotope ratio mass spectrometry (GC/C/IRMS). Using this novel enrichment technique, the present study extends measures of DHA turnover in the peripheral tissues, including red blood cells (RBC), perirenal adipose tissue (PRAT), muscle, heart, and skin. Mice were fed a low *δ* ^13^C diet (fish‐DHA control) for 3 months, then switched to either a high *δ* ^13^C treatment diet (algal‐DHA) or a very high *δ* ^13^C treatment diet (^13^C enriched‐DHA), while some remained on the fish‐DHA control diet as a reference group for the remainder of the study time course. In mice fed the algal and ^13^C enriched‐DHA diets, the RBC DHA half‐life was 22.8 and 19.5 days, the PRAT DHA half‐life was 6.0 and 8.2 days, the muscle DHA half‐life was 38.2 and 42.2 days, the heart DHA half‐life was 12.4 and 10.5 days, and the skin DHA half‐life was 13.6 and 13.0 days, respectively. Future studies could employ the ^13^C enrichment method to examine how DHA metabolism is altered in peripheral tissues according to genetics, stress, and development.

**Abbreviations**
ALA: alpha‐linolenic acid (18:3n‐3)
ANOVA: analysis of variance
BF₃: boron trifluoride
CSIA: compound‐specific isotope analysis
DHA: docosahexaenoic acid (22:6n‐3)
FA: fatty acid
FAME: fatty acid methyl ester
GC: gas chromatography
GC/C/IRMS: gas chromatography–combustion–isotope ratio mass spectrometry
GC/FID: gas chromatography–flame ionization detection
IRMS: isotope ratio mass spectrometry
mUr: MilliUrey (‰, per mil)
NEFA: non‐esterified fatty acid
PL: phospholipid
PRAT: perirenal adipose tissue
PUFA: polyunsaturated fatty acid
RBC: red blood cell
SE: standard error
TAG: triacylglycerol
*δ* ^13^C: carbon‐13 content

## 1 Introduction

Docosahexaenoic acid (DHA, 22:6n‐3) is an n‐3 polyunsaturated fatty acid (PUFA) that regulates important physiological processes across various tissues and organs, either directly or upon conversion to bioactive lipid mediators. Specifically, DHA is consumed through multiple pathways, including the enzymatic synthesis of bioactive metabolites such as oxylipins that regulate cell‐to‐cell communication, including inflammation, as well as mitochondrial β‐oxidation and non‐enzymatic autoxidation under oxidative stress (Klievik, Tyrrell, et al. 2023). Given DHA's broad physiological roles, understanding its turnover, encompassing both metabolism and replenishment, is essential for tracking its availability across tissues. DHA turnover involves the continuous release and replacement of DHA, primarily from phospholipid (PL) membranes and triacylglycerol (TAG) stores. In PL membranes, DHA is released by phospholipases and replaced with newly acquired DHA, often from the diet or synthesized from alpha‐linolenic acid (ALA). In TAG stores, DHA is mobilized through lipolysis, which breaks down TAGs into non‐esterified fatty acids (NEFAs) and is later replenished via re‐esterification with newly acquired DHA. Because DHA is metabolized and utilized as part of cellular processes, its consumption can be measured indirectly through the measurement of turnover rates, providing a useful indicator of tissue‐specific demand and utilization. Factors such as stress, environmental exposures, or genetic variation can alter metabolic demand, potentially affecting DHA consumption and tissue turnover.

Radiolabelled tracers and positron emission tomography imaging have been useful when studying tissue DHA kinetics, but they are expensive and relatively invasive. Our laboratory has developed an alternative, cost‐effective technique, called CSIA, that takes advantage of natural differences in carbon‐13 content (^13^C/^12^C ratio or *δ* ^13^C) of the food supply to better understand tissue DHA metabolism. CSIA can pinpoint the origin of a specific compound of interest since the carbon isotopic makeup of a molecule is conserved following incorporation from the diet (Lacombe et al. 2020). Importantly, natural variations in the photosynthetic pathways of C3 and C4 plants, along with marine fish sources, result in different isotopic signatures which can be used to measure tissue turnover rates (Lacombe et al. 2020; O'Leary 1981). The C3 photosynthetic pathway discriminates against carbon‐13 and, therefore, has low carbon‐13 in tissues (−20 to −32 milliUrey [mUr]; where 1 mUr equals 1 part per thousand (‰), or 0.1% ^13^C to ^12^C). The C4 photosynthetic pathway does not discriminate against carbon‐13 and, therefore, has higher carbon‐13 in tissues (−9 to −17 mUr) (Boutton 1991). Moreover, marine‐based aquatic organisms yield more intermediate carbon‐13 in fatty acids (−18 to −30 mUr) (Boutton 1991). Furthermore, due to lakes and rivers having less dissolved carbon pools, freshwater‐based aquatic phytoplankton appear to have less enriched *δ* ^13^C values than their marine counterparts, ranging from −42 to −26 mUr (Boutton 1991).

Previously, we applied CSIA to determine the half‐life of brain, liver, and plasma DHA in mice following a dietary switch experiment using different n‐3 PUFA sources of DHA in diets, each consisting of distinct carbon‐13 signatures: low *δ* ^13^C (fish‐DHA), high *δ* ^13^C (algal‐DHA), and a very high *δ* ^13^C (^13^C enriched‐DHA) to validate its utility in diet switch studies. The ^13^C enriched‐DHA treatment diet was formulated by enriching a fish‐DHA source with uniformly labeled ^13^C‐DHA (Klievik, Metherel, et al. 2023). Mice were fed a fish‐DHA diet (control) for 3 months, then switched to an algal‐DHA treatment diet, the ^13^C enriched‐DHA treatment diet, or they stayed on the control diet for the remainder of the study time course.

In mice equilibrated to the control diet and switched onto the ^13^C enriched‐DHA diet, the brain DHA half‐life was almost identical to the rate obtained after switching from the control diet to the algal‐DHA diet with no statistically significant differences. Additionally, there were no significant differences in liver and plasma tissues between the diet switches, validating the use of ^13^C‐enriched DHA diets for measuring tissue DHA turnover and half‐lives. Our research showed that DHA turnover in the brain of wild‐type mice is slower compared to the liver and plasma, where DHA is utilized more rapidly, reflecting the distinct metabolic roles and regulatory activity of DHA across tissues. Using this novel enrichment technique, the present study measures DHA turnover in RBC, PRAT, muscle, heart, and skin.

## 2 Materials and Methods

### 2.1 Materials

The fatty acid internal standard, docosatrienoic acid (22:3n‐3) ethyl ester, GC (gas chromatography) reference standard (GLC‐462), fish oil ethyl DHA, and microalgal ethyl DHA were purchased from NuChek Prep Inc. (Elysian, MN) as previously described (Klievik, Metherel, et al. 2023). Uniformly labeled ^13^C‐DHA methyl ester was purchased from Cambridge Isotope Laboratories Inc. Uniformly labeled ^13^C‐DHA was used to ensure that the isotopic labeling was consistent across all carbon atoms of the DHA molecule, allowing for accurate interpretation of the isotope ratios measured by CSIA. For isotopic analysis, the reference materials (USGS70, USGS71, USGS72) were obtained from Reston Stable Isotope Laboratory‐United States (Reston, VA). Boron trifluoride in methanol (14%) was purchased from Sigma‐Aldrich. All solvents used were American Chemical Society or high‐performance liquid chromatography (HPLC) grade and were purchased from either Millipore Sigma (Mississauga, ON, Canada) or Fisher Scientific (Ottawa, ON, Canada).

### 2.2 Animals

The University of Toronto Animal Ethics Committee approved the experimental animal protocol (protocol # 20012549), which was conducted in accordance with the policy and guidelines of the Canadian Council on Animal Care and the Regulations of Animals Research Act of Ontario. The animal facility maintained standard conditions in a temperature‐controlled (21°C) environment under a 12 h light/12 h dark cycle. Throughout the study, food and water were available *ad libitum*, and substantial care was taken to minimize animal suffering.

**FIGURE 1:**
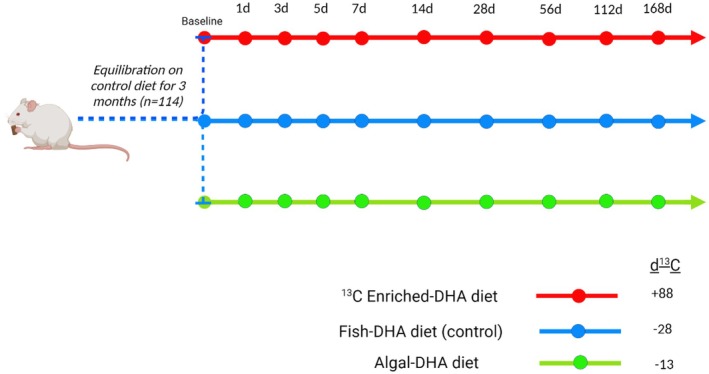
Diet switch experimental study design schematic. One hundred and fourteen 21‐day‐old male BALB/c pups were equilibrated to the fish‐DHA diet (control) for 3 months to establish a stable brain DHA isotopic signature prior to the diet switch. From the fish‐DHA equilibrated diet group, mice were stratified randomly onto either the algal‐DHA treatment diet, the ^13^C enriched‐DHA treatment diet, or they stayed on the control diet for the remainder of the study time course. Treatment and control mice (*n* = 4) were sacrificed at 1, 3, 5, 7, 14, 28, 56, 112, and 168 days post diet switch. A group of six mice from the equilibrated control diet was sacrificed prior to the diet switch for baseline measurements.

As previously described (Klievik, Metherel, et al. 2023), 114 twenty‐one‐day‐old BALB/c male pups were ordered (Charles River, Wilmington, MA), and upon arrival, acclimated for 1 week. The male pups were placed on the low *δ* ^13^C diet (fish‐DHA control) for 3 months to establish stable tissue DHA levels and isotopic signatures. Following 3 months of the fish‐DHA control diet, mice were randomized to one of three diets: (1) a high *δ* ^13^C treatment diet (algal‐DHA), (2) a very high *δ* ^13^C treatment diet (^13^C enriched‐DHA), or (3) they remained on the fish‐DHA control diet (Figure 1). Post diet switch, the mice were euthanized at 1, 3, 5, 7, 14, 28, 56, 112, and 168 days. A group of six mice from the equilibrated control diet was euthanized prior to the diet switch for baseline measurements. Blood was collected from the left ventricle prior to euthanasia by intracardiac perfusion under isofluorane anesthetic. PRAT, muscle, heart, and skin were collected, and along with the blood was flash frozen in liquid nitrogen, and stored at −80°C until biochemical processing.

### 2.3 Diets

Three DHA diets were formulated from the AIN‐93G diet (Dyets Inc., Bethlehem, PA), each containing DHA from different sources (fish‐DHA, algal‐DHA, and ^13^C enriched‐DHA) as previously described (Klievik, Metherel, et al. 2023). All three diets are isocaloric and contain by weight 10% fat, 60% carbohydrate, 20% protein, 5% insoluble fiber, and 5% vitamins/minerals/essential amino acids. Background oils were derived from safflower, fully hydrogenated coconut oils, and added oils (33.8%, 64.2%, and 2.0% by weight, respectively). Added oils were 2% fish oil DHA ethyl ester (Nu‐Chek Prep Inc.) for the fish‐DHA diet, 2% micro algae source DHA ethyl ester (Nu‐Chek Prep Inc.) for the algal‐DHA diet, and a mix of 99.75% fish oil DHA ethyl ester and 0.25% ^13^C‐DHA methyl ester (Cambridge Isotope Laboratories Inc.) for the ^13^C enriched‐DHA diet. The added oils for the fish‐DHA and algal‐DHA diets were sent to Dyets from the supplier for diet formulation. The added oils for the ^13^C enriched‐DHA diet were delivered and mixed at the University of Toronto, then sent to Dyets Inc. for diet formulation. The fatty acid compositions of experimental diets were measured in our lab by gas chromatography–flame ionization detection (GC/FID) in triplicate, and *δ* ^13^C‐DHA signatures of each diet were measured by gas chromatography combustions isotope ratio mass spectrometry (GC/C/IRMS), described previously (Klievik, Metherel, et al. 2023). The carbon isotope ratios of the fish, algal, and ^13^C enriched‐DHA diets were determined to be −28.2 ± 0.01, −13.2 ± 0.07, and + 87.8 ± 0.77 mUr, respectively.

### 2.4 Lipid Extraction and Transesterification

Total lipids were extracted from diets (fish‐DHA, algal‐DHA, and ^13^C enriched‐DHA) and tissues (PRAT, gastrocnemius muscle, heart, and abdominal skin) by modified methodologies adapted from Folch, Lees, and Sloane Stanley (Folch et al. 1957; Klievik, Metherel, et al. 2023). PRAT (~20 mg) was weighed into test tubes before lipid extraction. Muscle, heart, and skin tissue were pulverized by a tissue pulverizer (Cole‐Parmer; item no. RK‐36903‐05) in the presence of liquid nitrogen; the pulverizer was re‐chilled in liquid nitrogen for 3 min prior to every sixth tissue, and (~50, ~50, and ~20 mg, respectively) were weighed into test tubes. For PRAT, muscle, heart, and skin, total lipids were extracted with 2:1 chloroform:methanol with a known mass of docosatrienoic acid ethyl ester internal standard (22:3n‐3; NuChek Prep Inc.) for fatty acid quantification. Tissues were left exposed to extraction solvents at room temperature for 48 h. Following the 48‐h period, 1.75 mL of 0.88% potassium chloride aqueous buffer was added to separate lipid‐containing and aqueous phases. The lipid‐containing chloroform phase was isolated, and an aliquot of tissue lipid extract was dried under a stream of nitrogen and used for transesterification. All samples were trans‐esterified using methods adapted from Morrison and Smith (Morrison and Smith 1964). Transesterification to fatty acid methyl esters (FAMEs) was performed by the addition of 1 mL of 14% boron trifluoride (BF_3_) in methanol (MilliporeSigma, Burlington, MA) and 0.3 mL hexane and heating in an oven for 1 h at 100°C. After samples were cooled to room temperature, 1 mL of milliQ H_2_O and 1 mL of hexane was added. The samples were vortexed, centrifuged at 500 × g, and the upper hexane layer was separated. This layer was dried under nitrogen, reconstituted in 100 μL of heptane, and stored in GC vials for analysis.

For RBC analysis, ~25 μL of each sample was pipetted into test tubes using cut tips to facilitate easier pipetting, after which 1 mL 14% BF_3_ and 0.3 mL hexane containing 5 μg of 22:3n‐3 were added and placed on a heat block at 95°C for 1 h. After cooling, similarly to tissues mentioned above, 1 mL of hexane and 1 mL of water were added to the samples. They were vortexed, centrifuged at 500 × g, and the top hexane layer was isolated, dried under nitrogen, reconstituted in 75 μL of heptane, and stored in GC vials until analysis.

### 2.5 FAME Quantification

Fatty acid methyl esters were quantified via GC/FID using a Varian 430 GC/FID (Scion) as previously described (Klievik, Metherel, et al. 2023; Lacombe et al. 2020). A DB‐FFAP 30 m × 0.25 mm i.d. × 0.25 μm film thickness, nitroterephthalic acid modified, polyethylene glycol, capillary column was used (Agilent; item no. 122‐3232). The column oven program was initially set at 50°C for 1 min, increased at a rate of 30°C/min to 130°C, increased at 10°C/min to 175°C, increased at 5°C/min to 230°C, and held for 9.5 min, and finally increased to 240°C at a rate of 50°C/min and held for 11.13 min, totaling 40 min. Chromatogram peaks were identified by comparing retention times to the GLC‐462 external reference standard (NuChek Prep Inc.) and quantified by comparing the peak area to that of the internal standard. After GC/FID quantification, recapped vials were then stored at −80°C until GC/C/IRMS analysis.

### 2.6 Compound Specific Isotope Analysis

RBC, PRAT, muscle, heart, and skin *δ* ^13^C of FAMEs were determined by GC/C/IRMS. FAMEs (1–3 μL) were injected onto a 100 m, 0.25 mm i.d., 0.20 μm *d*_f_; Supelco SP‐2560 (by Sigma‐Aldrich) capillary column in a Thermo Scientific Trace 1310 GC interfaced to a MAT 253 IRMS (Thermo Finnigan MAT, Bremen, Germany) via a GC Iso Link II combustion interface (Thermo Scientific) using a TriPlus RSH autosampler (Thermo Fisher Scientific). Complete separation was achieved with the following program: initial temperature of 60°C with an immediate ramp of 15°C/min to 180°C with no hold, followed by a 1.5°C/min ramp to 240°C with an 18‐min hold for a total run time of 66 min. The carrier flow rate was set to 1.2 mL/min, yielding baseline resolutions of analyte peaks of interest. As a result of helium carrier gas, the GC effluent was swept to a Thermo Fisher Scientific GC Iso Link II combustion interface at 1000°C, with nickel and copper catalysts, and the MAT 253 IRMS (Thermo Fisher Scientific) was interfaced via a ConFlo IV (Thermo Fisher Scientific) continuous‐flow interface. Prior to entering the IRMS ion source (Dupont, Wilmington, DE), the CO_2_ gas produced by quantitative combustion of isolated analytes was dried by flowing gas through a Nafion dryer.

### 2.7 Carbon Isotope Ratio Normalization and Methyl Corrections

Using multipoint linear normalization similar to previously described methods (Lacombe et al. 2017; Paul et al. 2007), all carbon isotope ratios were normalized with consensus‐validated 20‐carbon FAME reference material USGS70, USGS71, and USGS72 (Reston Stable Isotope Laboratory). In USGS70, USGS71, and USGS72, consensus‐derived carbon isotope ratios are −30.53, −10.50, and −1.54, respectively and are expressed relative to Vienna Peedee Belemnite on a scale normalized to primary reference materials NBS 19 and LSVE (Schimmelmann et al. 2016). Under similar analytical conditions to the analyzed samples, reference materials were injected twice throughout each batch of samples analyzed by GC/C/IRMS and elemental analysis/IRMS. Based on the measured and accepted ^13^C values of reference materials, linear regression lines were used to generate normalizing equations for each batch of samples. *R* ^2^ values for all equations were 0.9998. CO_2_ gas produced through the combustion of sample material or specific organic analytes is measured by IRMS, which cannot distinguish between derivatized carbon units added to fatty acids during transmethylation. By using the following mass balance equation, methyl corrections were performed to account for the contribution of derivatized carbon units to measured carbon isotope ratios, in order to report true values.

(1) $$n_{\text{FAME}}\delta^{13}C_{\text{FAME}}=\delta^{13}C_{\mathrm{ME}}+n_{\mathrm{FA}}\delta^{13}C_{\mathrm{FA}}$$

*δ* ^13^C is defined as the isotope ratio of ^13^C to ^12^C. The subscripts FAME, ME, and FA are the measured carbon isotope ratios of FAMEs, methyl groups, and fatty acids, respectively, where n refers to the number of moles of carbon in the corresponding FAMEs and fatty acids. nFAME and nFA are equal to 23 and 22, respectively, for methyl corrections of measured DHA carbon isotope ratios. In each batch of samples, the carbon isotope ratios of the methyl group were determined using methylated and free heptadecanoic acid standards (Nu‐Chek Prep Inc.). The carbon isotope signature of the methyl group was −40.5 mUr.

### 2.8 Statistics and Calculations

All reported data are expressed as means ± SE to show how much the mean DHA values vary across tissues and timepoints. The sample size ranged from three to four mice per timepoint throughout the study. A two‐way analysis of variance (ANOVA) was used to compare RBC, PRAT, muscle, heart, and skin DHA concentrations in mice after the diet switch using the interaction of time and diet group at *p* < 0.05. When an interaction was observed, a one‐way ANOVA was performed, with significance determined at *p* < 0.05. Statistical significance for half‐life calculations was determined by non‐overlapping 95% confidence intervals.

Tissue *δ* ^13^C‐DHA signatures from mice following the diet switch were plotted over time and modeled with a one‐phase decay function to generate a curve of best fit:

(2) $$y={\left(y_{0}-\text{plateau}\right)}^{-\mathrm{kx}}+\text{plateau}$$

$y_{0}$ defines the best‐fit curve at time zero, plateau defines the value of the best‐fit curve at an infinite time, and *k* defines the decay constant. Incorporation half‐lives for tissue *δ* ^13^C‐DHA signatures were determined from the best‐fit curves using the following formula:

(3) $$t_{1/2}=\frac{\ln\left(2\right)}{k}$$

Based on a one‐phase decay function, k is the decay constant. The following formula was used to calculate the rate of loss of DHA from the tissue DHA pool, $J_{\text{out}}$ (μmol/g tissue/day) (DeMar et al., DeMar Jr. et al. 2004; Lin et al. 2015; Rapoport 2001):

(4) $$J_{\text{out}}=\frac{0.693C_{\mathrm{FA}}}{t_{1/2}}$$

$C_{\mathrm{FA}}$ is the baseline DHA concentration from the given tissue lipid pool and $t_{1/2}$ is the corresponding experimentally derived half‐life.

To approximate the net incorporation rate of DHA, the rate of loss of DHA from the DHA tissue pool, *J*_out_, must be assumed to be equal when the net incorporation rate of DHA and the net loss rate of DHA are at steady state (Lacombe et al. 2020).

GraphPad Prism, Version 9 (GraphPad Software, San Diego, CA, USA) was used to perform the two‐way ANOVA, one‐way ANOVA, and one‐phase exponential decay function tests.

## 3 Results

### 3.1 Fatty Acid Concentration of DHA in RBC, PRAT, Muscle, Heart, and Skin After Diet Switch

**FIGURE 2:**
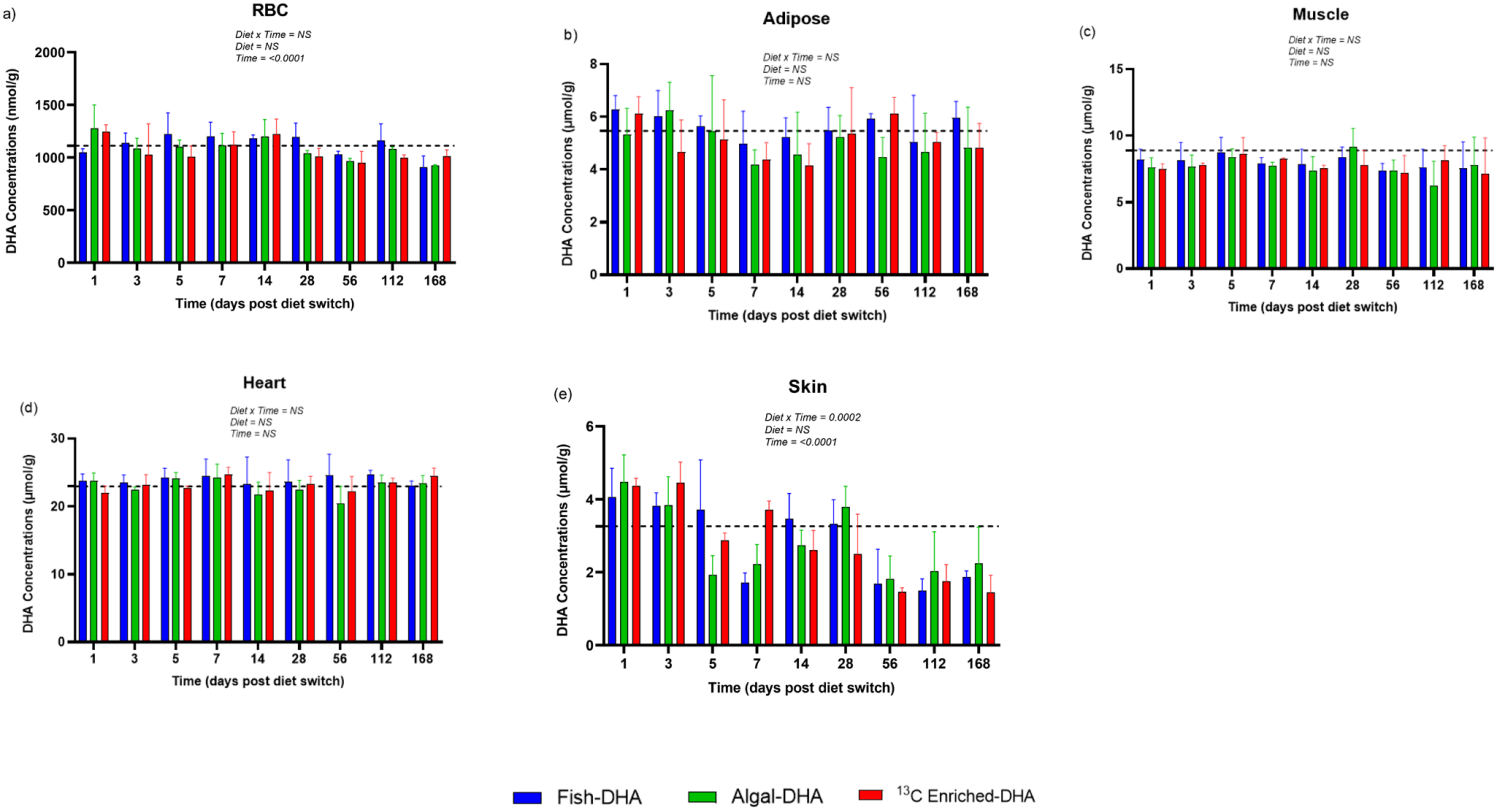
RBC (a), adipose (b), muscle (c), heart (d), and skin (e) DHA concentrations (bars) following the diet switch from mice equilibrated to the control (fish‐DHA) diet. Data are shown as means ± SEs and were compared by two‐way ANOVA for the interaction of time and diet (*n* = 3–4 per timepoint). *p* < 0.05 was considered statistically significant. Baseline DHA concentrations are represented by the dashed line with the black band. DHA, docosahexaenoic acid; NS, not significant.

RBC, PRAT, muscle, heart, and skin were collected from mice consuming the fish, algal, and ^13^C enriched‐DHA diets throughout the time course of 168 days (Figure 1). DHA concentrations of RBC, PRAT, muscle, heart, and skin are presented in Figure 2A–E, respectively.

After the diet switch, throughout the time course, a significant effect of time (*p* < 0.0001) was observed for RBC DHA concentrations (Figure 2A). The DHA RBC baseline concentration had a value of ~1111 nmol/g and had a range between ~900 and 1300 nmol/g. PRAT DHA concentrations had no significant interaction of diet and time, and neither diet nor time affected DHA concentrations (Figure 2B). The DHA baseline concentration for the PRAT was ~5.5 μmol/g and had a range between ~4.0 and 6.0 μmol/g. Muscle DHA concentrations had no interaction of diet or time, and neither diet nor time affected DHA concentrations. The muscle DHA baseline concentration was ~8.9 μmol/g and had a range between ~6.0 and 9.0 μmol/g (Figure 2C). Heart DHA concentrations had no interaction of diet or time, and neither diet nor time affected DHA concentrations. The heart DHA baseline concentration was 23.0 μmol/g and had a range between 20.0 and 25.0 μmol/g (Figure 2D). A significant effect of time (*p* < 0.0001) and time by diet interaction (*p* = 0.0002) was observed for skin DHA concentrations (Figure 2E). The DHA skin concentrations had a range between ~1.5 and 4.5 μmol/g, with a baseline DHA value of ~3.3 μmol/g. Even though DHA concentrations remained relatively stable in most tissues over time, using our method, we were able to measure how fast DHA was being used and replaced.

### 3.2 Carbon Isotopic Analysis for RBC, PRAT, Muscle, Heart, and Skin

In control mice equilibrated to the fish‐DHA diet over 3 months, we were able to establish a stable DHA isotopic signature of −28.3 ± 0.28, −29.6 ± 0.36, −29.1 ± 0.13, −27.1 ± 0.12, and −29.9 ± 0.86 mUr for the RBC, PRAT, muscle, heart, and skin, respectively.

**FIGURE 3:**
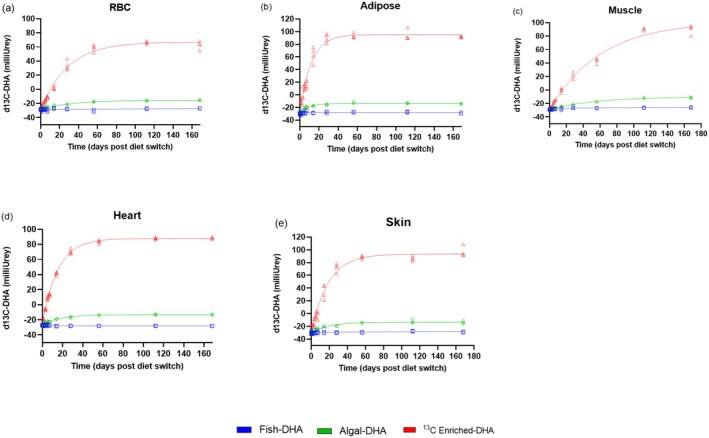
Change in RBC (a) adipose (b), muscle (c), heart (d), and skin (e) *δ* ^13^C‐_DHA_ signatures following the diet switch from mice equilibrated to the control (fish‐DHA) diet. Data are shown as individual data points and modeled with a one‐phase decay function (*n* = 3–4 mice per timepoint). DHA, docosahexaenoic acid.

**FIGURE 4:**
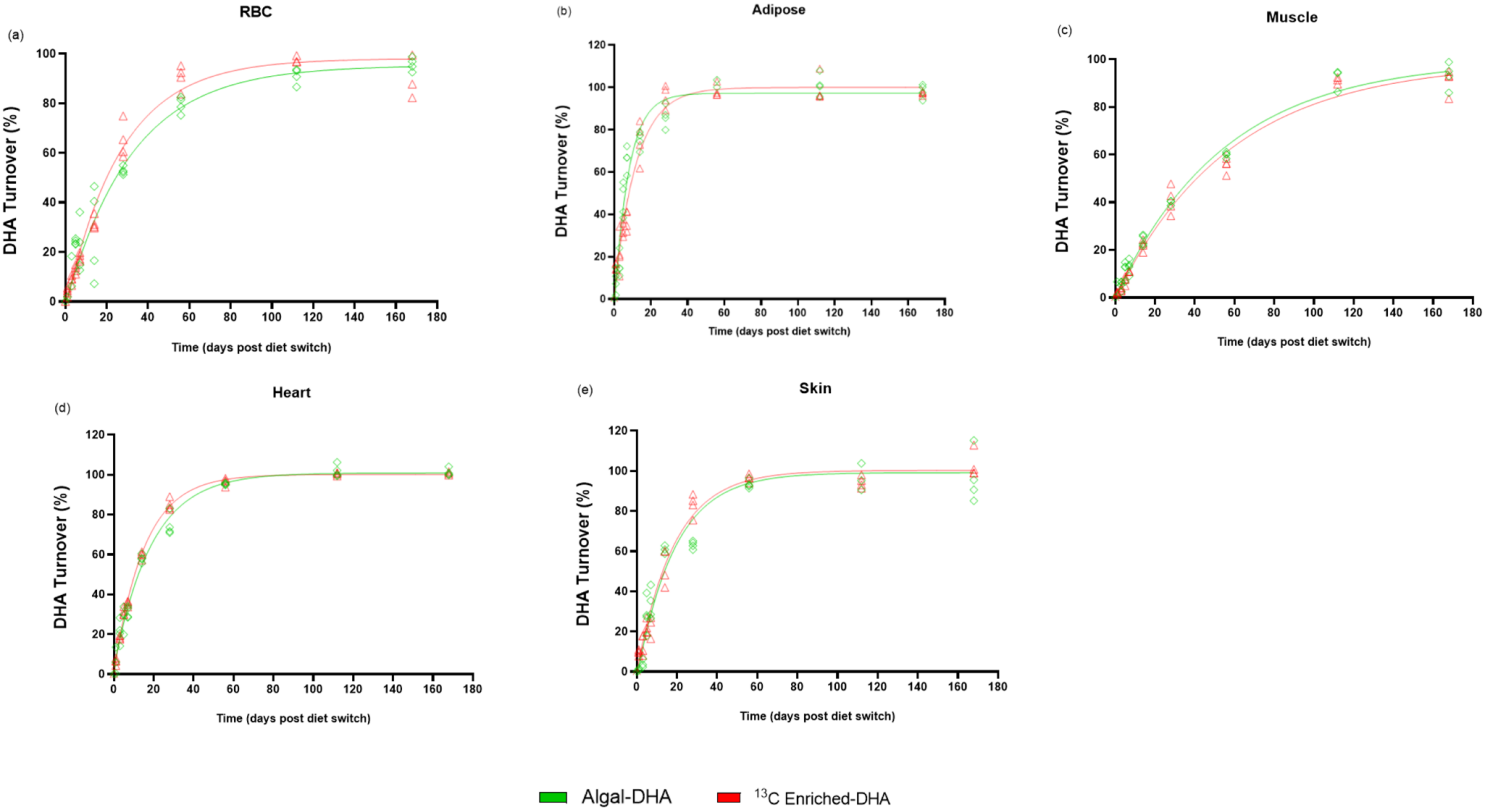
Percent DHA turnovers in RBC (a), adipose (b), muscle (c), heart (d), and skin (e) tissues following the diet switch from mice equilibrated to the control (fish‐DHA) diet. Data are shown as individual data points (*n* = 3–4 mice per timepoint). DHA, docosahexaenoic acid.

Changes in RBC, PRAT, muscle, heart, and skin δ^13^C‐DHA signatures following the diet switch are displayed in Figure 3, and their respective percent DHA turnovers are displayed in Figure 4. Over the 168‐day period, RBC, PRAT, muscle, heart, and skin DHA signatures were measured and fitted to a one‐phase decay function to model DHA tissue turnover.

Kinetic parameters from best‐fit curves of *δ* ^13^C‐DHA signatures in response to the diet switch after equilibration of the fish‐DHA (control) diet are displayed in Table 1. The RBC DHA half‐life was 22.8 days [95% CI, 17.7–29.7] in mice fed the algal‐DHA diet and 19.5 days [95% CI, 17.5–21.8] in mice fed the ^13^C enriched‐DHA diet. When comparing DHA half‐lives in the blood, the DHA half‐life in RBC is approximately 3–4.9‐fold slower than in plasma, as previously reported (Klievik, Metherel, et al. 2023). The PRAT DHA half‐life was 6.0 days [95% CI, 4.9–7.4] in mice fed the algal‐DHA diet and 8.2 days [95% CI, 7.1–9.4] in mice fed the ^13^C enriched‐DHA diet. The muscle DHA half‐life was 38.2 days [95% CI, 33.0–44.8] in mice fed the algal‐DHA diet and 42.2 days [95% CI, 37.0–48.6] in mice fed the ^13^C enriched‐DHA diet. Comparing muscle to PRAT, DHA in muscle tissue has a turnover rate that is approximately 5–6.4‐fold slower than in PRAT. The heart DHA half‐life was 12.4 days [95% CI, 10.8–14.3] in mice fed the algal‐DHA diet and 10.5 days [95% CI, 10.1–11.0] in mice fed the ^13^C enriched‐DHA diet. The turnover of DHA in the heart is 1.3–2‐fold slower than PRAT and 3 to 4‐fold faster than muscle. The skin DHA half‐life was 13.6 days [95% CI, 10.0–18.6] in mice fed the algal‐DHA diet and 13.0 days [95% CI, 11.4–14.8] in mice fed the ^13^C enriched‐DHA diet, which is similar to the heart in terms of turnover rate, with skin turnover being about 1.6–2.3‐fold slower than in PRAT and 3‐fold faster than in muscle. Based on overlapping 95% confidence intervals, there were no statistical differences in RBC, PRAT, muscle, heart, and skin DHA half‐lives between diet switch groups. Half‐lives and kinetic parameters could not be established for the control mice maintained on the fish‐DHA diet because *δ* ^13^C‐DHA remained unchanged throughout the time course. This group was not adequately modeled by a one‐phase decay function.

**TABLE 1:** Kinetic parameters from best‐fit curves of *δ* ^13^C‐DHA signatures in response to the diet switch after equilibration on the fish‐DHA control diet.

Kinetic parameters from best‐fit curves of δ^13^C−DHA signature response to dietary switch after equilibration of fish−DHA control diet						
Tissue	Diet	Y_0_, mUr	Plateau, mUr	*k*,days	*t*_1/2_, days (95% CI)	*J*_out_ (μmol/g/day)
RBC	Algal‐DHA	−28.44	−15.44	0.03	22.8 (17.7−29.7)	0.03
	^13^C Enriched‐DHA	−30.07	67.04	0.04	19.5 (17.5−21.8)	0.04
Adipose	Algal‐DHA	−29.91	−13.74	0.12	6.0 (4.9−7.4)	0.64
	^13^C Enriched‐DHA	−28.61	95.19	0.08	8.2 (7.1−9.4)	0.46
Muscle	Algal‐DHA	−28.89	−10.15	0.02	38.2 (33.0−44.8)	0.16
	^13^C Enriched‐DHA	−29.06	101.90	0.02	42.2 (37.0−48.6)	0.15
Heart	Algal‐DHA	−26.76	−13.14	0.06	12.4 (10.8−14.3)	1.29
	^13^C Enriched‐DHA	−26.64	87.80	0.07	10.5 (10.1−11.0)	1.52
Skin	Algal‐DHA	−30.27	−13.41	0.05	13.6 (10.0−18.6)	0.17
	^13^C Enriched‐DHA	−30.47	93.40	0.05	13.0 (11.4−14.8)	0.18

*Note:* A one‐phase decay function was utilized to generate kinetic parameters based on best−fit curves generated through δ^13^C DHA signatures. Significance for half‐lives were determined by non‐overlapping 95% confidence intervals.

Abbreviations: CI, confidence interval; $J_{\text{out}}$, synthesis rate; *k*, decay constant; Plateau, value of the best‐fit curve at an infinite time; $t_{1/2}$, half‐life; $y_{0}$, best‐fit curve at time zero.

### 3.3 The Rate of Loss of DHA (*J*_out_)

Because DHA concentrations in the RBC, PRAT, muscle, heart, and skin were relatively stable over time, total lipid *J*_out_ values were calculated for each tissue (Table 1). Estimates of the daily synthesis rates or daily rate of loss (*J*_out_) are determined by using Equation (4) (see Section 2.8). The rate of DHA out of a tissue is approximately the rate of incorporation of DHA into a tissue, given that the DHA tissue pool sizes remain constant. The heart displays the fastest DHA loss rates, being 7.6–8.4‐fold faster than the skin and 8.0–10‐fold faster than muscle. The rate of DHA loss in PRAT tissue is 3–4‐fold faster than in muscle and 2–3‐fold slower than in the heart. Skin and muscle have similar DHA *J*_out_ values, though skin is slightly faster than muscle by 1.1–1.2‐fold.

## 4 Discussion

DHA tissue turnover has been studied in monkey RBC and mouse adipose tissue (Connor et al. 1990; Lacombe et al. 2020). In this paper, we utilize a novel ^13^C enrichment technique to validate existing literature values for RBC and adipose and, for the first time, measure DHA turnover in muscle, heart, and skin.

### 4.1 RBC

In mice equilibrated to the control diet and switched to the algal‐DHA diet or the ^13^C enriched‐DHA diet, the DHA half‐life in the RBC was determined to be 22.8 and 19.5 days, respectively (Table 1). These results closely resemble those from a study of rhesus monkeys fed a DHA‐rich fish oil diet for up to 129 weeks, which estimated a DHA half‐life of 21 days, representing the time required to reach half of the final steady‐state DHA concentration (Connor et al. 1990). It is important to note that, while the results are consistent with the present study, there are differences in both methodology and species. Furthermore, RBC fatty acids provide a more accurate reflection of long‐term intake compared to plasma fatty acids due to their reduced sensitivity to recent intake and slower turnover rate (Sun et al. 2007). This observation supports our findings, as the DHA half‐life in RBC we measured is longer than the 4.7–6.4‐day turnover rate of DHA in plasma. RBC are crucial for oxygen transport throughout the body, and they rely on fatty acids like DHA to maintain their structure and function. In conditions characterized by chronic inflammation, whether due to hematological disorders (e.g., anemia, sickle cell disease) or non‐hematological disorders (e.g., obesity, kidney disease), DHA may be increasingly utilized to preserve RBC function and produce bioactive lipid mediators to help resolve inflammation, influencing DHA turnover. Future research in mouse disease models could apply the ^13^C enrichment method to track DHA turnover in RBC, providing valuable insight into how DHA metabolism may be altered in these conditions driven by environmental stressors, genetic predispositions, or disease‐related inflammation.

### 4.2 PRAT

The half‐life of DHA in the PRAT was determined to be 6.0 and 8.2 days after being switched on to the algal‐DHA and ^13^C‐enriched DHA diet, respectively (Table 1). These results were broadly similar to a previously published study by Lacombe et al., where the half‐life of DHA in the adipose (epididymal) was determined to be approximately 5.4 days upon DHA feeding (Lacombe et al. 2020). Lacombe et al. similarly applied CSIA to determine the half‐life of adipose DHA in mice following a diet switch experiment using different n‐3 PUFA sources (ALA and DHA). However, this model was limited by (1) not including earlier timepoints within the first week, which missed the window for DHA turnover in the adipose and (2) the lack of steady‐state DHA pool sizes in peripheral tissues limiting accurate DHA turnover estimates. In the present study, the addition of earlier timepoints (days 1, 3, and 5), and using a diet switch containing different n‐3 PUFA sources of DHA (rather than different n‐3 PUFA) more accurately captured the turnover of DHA in the adipose tissue (Figure 4b). Adipose tissue stores fatty acids, including DHA, which are released into circulation to meet energy demands, such as during exercise. In stress‐related conditions like obesity and metabolic syndrome, DHA is converted into bioactive lipid mediators to help regulate inflammation. However, in these conditions, the production of DHA‐derived mediators is often impaired (Fisk et al. 2022), reducing DHA utilization and limiting its ability to manage inflammation. As a result, this contributes to metabolic dysfunction, including increased lipolysis, insulin resistance, and elevated NEFAs. Future studies using the ^13^C enrichment method could track DHA turnover in adipose tissue to better understand how stressors such as exercise, obesity, and insulin resistance affect DHA metabolism and turnover using mouse models.

### 4.3 Muscle

The half‐life of DHA in the muscle was determined to be 38.2 and 42.2 days after being switched on to the algal‐DHA and ^13^C‐enriched DHA diet, respectively (Table 1). Since this is the first study investigating DHA turnover in muscle, research in this area is limited. However, studies by DeMots et al., Miller et al., and Arneson et al. investigated carbon turnover rates using bulk stable carbon analysis in muscle tissue of various mouse species under different dietary conditions, with findings relevant to the present study (Arneson 2006; DeMots 2010; Miller 2008). DeMots et al. conducted a 168‐day diet‐switch experiment in white‐footed mouse quadriceps transitioning from a harlan rodent chow diet to a corn‐based diet (diet switch 1), or they transitioned from a corn‐based diet to the harlan diet (diet switch 2). The calculated half‐lives for carbon turnover were 29.6 days during the first diet switch and 30.1 days during the second switch (DeMots 2010). Miller et al. examined carbon turnover in leg muscle tissue of wild deer mice switched to a control diet (deplete in carbon‐13), revealing a half‐life of 18.7 days (Miller 2008). Arneson et al. examined carbon turnover in the triceps muscle of BALB/c mice through short‐ and long‐term diet switch studies, where mice were switched from a control diet to a beet sucrose diet, resulting in carbon half‐lives of 23.1 days in the short‐term study and 18.2 days in the long‐term study (Arneson 2006). Although our study's results are largely consistent, minor variations in tissue half‐lives may be due to differences in mouse species used, the specific muscle tissues analyzed (e.g., leg [quadriceps] vs. arm [triceps]), and the measurement of carbon turnover in the muscle tissue compared to DHA turnover. The muscle DHA half‐life was the longest among the tissues analyzed. The longer DHA half‐life in muscle suggests that, under non‐stressful conditions, muscle tissue may act as a stable reservoir for DHA, supporting muscle integrity over time. Skeletal muscle is highly responsive to physiological stressors such as exercise, injury, and metabolic dysfunction, which trigger localized inflammation regulated by lipid mediators (Signini et al. 2020). For example, DHA‐derived resolvins and protectins can support resolution of inflammation and enhance muscle regeneration following injury (Jannas‐Vela et al. 2023), which may alter DHA turnover in muscle due to increased demand. By applying the ^13^C enrichment technique, future research could track DHA turnover in muscle during periods of stress, such as exercise, as well as developmental factors like aging and muscle‐wasting conditions such as sarcopenia.

### 4.4 Heart

The half‐lives of DHA in the heart were found to be 12.4 and 10.5 days after switching to the algal‐DHA and ^13^C‐enriched DHA diets, respectively (Table 1). As this is the first study to investigate DHA turnover in the heart, research in this area remains limited. However, these findings are consistent with the work of Arneson et al., who examined carbon turnover in the heart of BALB/c mice, reporting a turnover rate of 13.9 days following a short‐term diet switch (Arneson 2006). Slight differences between these studies may be attributed to factors such as the use of a different mouse strain and the measurement of carbon turnover rather than DHA directly in the tissues. Given the heart's high metabolic rate, constant contraction, and rapid blood flow to organs and tissues, these factors contribute to its elevated *J*_out_, making it the tissue with the greatest DHA loss as DHA is continuously incorporated and removed through the high blood flow. Since the heart is highly dependent on fatty acids as its primary fuel source under normal conditions, cardiovascular disease (CVD) often leads to increased inflammation and oxidative stress, which can disrupt the heart's metabolic processes. Oxylipins derived from 20‐ and 22‐carbon n‐3 PUFA, such as eicosapentaenoic acid (EPA) and DHA, are known for their anti‐inflammatory, anti‐aggregatory, and vasodilatory effects (Caligiuri et al. 2017). In response to CVD, the heart may require more DHA to modulate inflammation and reduce oxidative stress, potentially altering DHA turnover in cardiac tissue. Future research using ^13^C enrichment techniques could track DHA turnover in the heart, investigating how genetic predispositions to heart disease or lifestyle factors such as diet affect DHA metabolism. This approach could reveal how DHA turnover differs in response to both genetic and environmental stressors.

### 4.5 Skin

In the skin, the half‐life of DHA in mice switched on to the algal‐DHA diet or the ^13^C enriched‐DHA diet was determined to be 13.6 and 13.0 days, respectively (Table 1). Similarly to muscle and heart DHA turnover, research on DHA turnover in the skin is also limited, with carbon turnover rates being the closest comparison. Browning et al. investigated carbon turnover rates in bottlenose dolphin skin during two dietary switches (Diet switch 1: saury/atlantic mackerel to capelin/horse mackerel; Diet switch 2: capelin/horse mackerel to saury/atlantic mackerel), finding carbon half‐lives of 13.9 and 22.1 days for the first and second switches, respectively (Browning et al. 2014). Gimenez et al. conducted the longest controlled feeding experiment on bottlenose dolphins (350 days) to evaluate carbon turnover rates in the skin through a dietary switch where they were fed sprat/herring and then switched to sprat/capelin, estimating a carbon half‐life of 24.2 days (Giménez 2016). Similarly, Alves‐Stanley et al. looked at carbon turnover in the skin of manatees that were transitioning from a diet of aquatic forage to terrestrial forage, finding mean carbon half‐lives of 53 and 59 days depending on the diet switch (Alves‐Stanley and Worthy 2009). The slower turnover rate in manatees compared to dolphins observed could be attributed to the manatees' epidermis, which exhibits hyperkeratosis, leading to thickened skin and a delayed replacement of keratin, therefore contributing to the extended isotopic turnover in their skin. Therefore, the skin DHA turnover rates observed in our study may not be directly comparable to the carbon turnover rates in manatees but are broadly similar to those reported in dolphins. Notable differences may be due to variations in species, areas of skin biopsies analyzed, and similarly to muscle, the distinction between measuring carbon turnover versus DHA turnover. The epidermis serves as the body's first line of defense, protecting against harmful external factors such as ultraviolet (UV) light, radiation, and dryness (Harauma et al. 2024). Skin conditions, often linked to increased inflammation, can alter DHA demand and turnover. N‐3 PUFA have been shown to alleviate inflammatory skin diseases like atopic dermatitis, psoriasis, and even skin cancer in human and mouse models (Balic et al. 2020; Thomsen et al. 2020) and reduce skin inflammation by altering epidermal lipid mediators and lowering proinflammatory N‐acyl ethanolamines (Kendall et al. 2019). Using the ^13^C enrichment method, future studies could track DHA turnover in the epidermis to assess how genetic conditions (e.g., eczema, psoriasis) and environmental stressors (e.g., sunburn, UV‐induced skin cancer) affect DHA metabolism, as well as how aging influences turnover.

Based on our results in peripheral tissues, DHA turnover in adipose tissue and liver (as shown in our earlier study (Klievik, Metherel, et al. 2023)) appears to be more dynamic, with faster turnover rates compared to slower DHA turnover rates in tissues such as muscle, skin, and RBC. These findings suggest that the liver and adipose tissue may serve as more rapid sources of DHA. However, due to the ongoing exchange between tissue pools and TAG stores, it remains challenging to determine the precise contribution of each source. Future studies using targeted approaches, such as expressing the Fat‐1 gene specifically in adipose tissue (Giuliano et al. 2018), could help clarify the relative contribution of adipose‐derived DHA.

As previously mentioned (Klievik, Metherel, et al. 2023), a limitation of the study was that the fish‐DHA control and algal‐DHA diets used ethyl ester DHA, while the ^13^C‐enriched DHA diet used methyl ester DHA. Although no studies directly compare the absorption of ethyl versus methyl esters, research indicates that ethyl esters are absorbed more slowly than TAG due to the fatty acid‐ethanol bond being much more resistant to pancreatic lipase than the glycerol backbone in TAG (Yang et al. 1990a, 1990b). This difference might have led to slight variations in turnover rates between the algal and ^13^C‐enriched DHA groups when mice switched diets. Another limitation is that mice were not fasted before being sacrificed; therefore, some of the carbon isotope ratio measurements in tissues with shorter half‐lives (PRAT) may have been affected by the postprandial circulating TAG. There were some variations in carbon isotopic ratios in the PRAT tissues on days 3, 5, and 7. In future studies investigating adipose fatty acid turnover, animals should be fasted before sample collections to limit variations in carbon isotope ratios resulting from recently consumed diets. Furthermore, variations in carbon isotopic ratios and adipose concentration throughout the time course might have resulted from variations in food consumption. Future research should include monitoring food intake and mouse weight. Another limitation of the diet switch was the location of adipose tissue collection in each mouse ‐PRAT. Although there were no significant differences in the PRAT DHA concentrations across time, the PRAT DHA concentrations still varied slightly, as shown in Figure 2B. Despite this region being difficult to access and limited in size, PRAT undergoes an unusual progressive transition from brown adipose tissue into white adipose tissue after birth (Shi et al. 2012). In fetuses and newborns, brown adipocytes are the majority of PRAT, while white adipocytes compose only the thin outermost layer. Researchers found that as adult mice age, very few brown adipocytes persist within perirenal white adipose tissues (Huang et al. 2020). Since PRAT constitutes a combination of uneven white and brown adipose tissues, this may explain the variation in DHA concentrations in adipose tissues throughout time. Future studies investigating adipose tissue could harvest tissues from epididymal adipose tissues since the depot is bigger and easier to access (Huang et al. 2020). Lastly, this study is a secondary analysis of data from an experiment that was originally powered to estimate the half‐lives of DHA in the brain. As such, the sample size was determined based on brain tissue outcomes, and no separate power calculation was performed for peripheral tissues.

In summary, this study extends our previously validated ^13^C enrichment method to measure DHA turnover in peripheral tissues beyond the brain (Klievik, Metherel, et al. 2023). In prior work, brain DHA half‐life estimates using our method aligned similarly to those generated with other methods supporting the reliability of our approach. Here, for the first time using the ^13^C enrichment method, we report distinct half‐lives in RBC, PRAT, muscle, heart, and skin, revealing tissue‐specific differences in DHA consumption. Notably, turnover was fastest in PRAT and slowest in muscle. Future studies will use this ^13^C enrichment method in mouse models to explore how DHA metabolism is influenced by stress, development, and genetics.

## Author Contributions

B.J.K. and R.P.B. conceived and designed the study. B.J.K. wrote the first draft of the manuscript; B.J.K., A.H.M., and R.V. carried out the research. B.J.K. analyzed the data. All authors contributed to and approved the final draft of the manuscript.

## Conflicts of Interest

R.P.B. has received industrial grants, including those matched by the Canadian government, and/or travel support from Arctic Nutrition, Bunge Ltd., DSM, The Dairy Farmers of Canada, Mead Johnson, Natures Crops International, Nestec Inc., Pharmavite, and Sansero Life Sciences Inc. R.P.B. has served as a consultant to Bunge Ltd., Fonterra, and Red Abbey Labs. Moreover, R.P.B. was on the executive committee of the International Society for the Study of Fatty Acids and Lipids and held a meeting on behalf of fatty acids and cell signaling, both of which rely on corporate sponsorship. R.P.B. has given expert testimony in relation to supplements and the brain. Adam H. Metherel is on the Board of Directors of the International Society for the Study of Fatty Acids and Lipids, is a Science Advisor for Benexia and Natures Crops International, and is a co‐applicant on a joint government/industry‐funded research grant with Natures Crops International.

## Data Availability

All datasets generated during and/or analyzed during the current study are available from the corresponding author on reasonable request.
